# Supplementary figures and images for: Context-dependent requirement of G protein coupling for Latrophilin-2 in target selection of hippocampal axons
Source: eLife. 2023 Mar 20;12:e83529. doi: 10.7554/eLife.83529 (PMC10118387; doi:10.7554/eLife.83529)

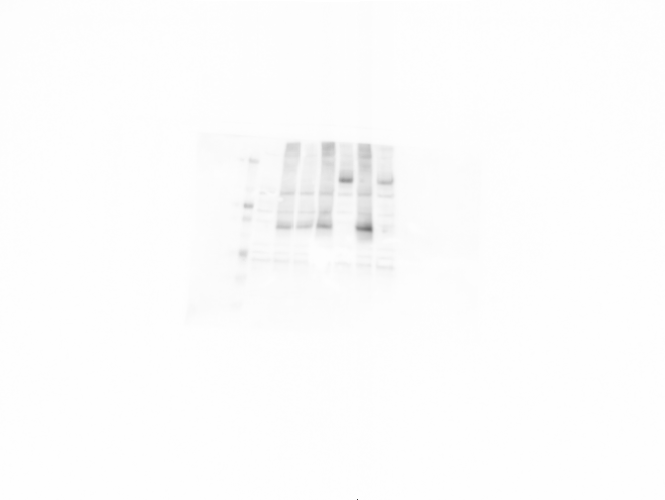

Supplement: Figure 2—source data 1. — Primary antibody against Flag (1:500, ThermoFisher, PA1-984B), secondary antibody anti-rabbit HRP (1:10,000, ThermoFisher, Cat #31458). The same blot was used in Figure 3B and Figure 4A. [file elife-83529-fig2-data1.zip › Figure 2-source data 1/Figure 2 -Source 1.TIF]

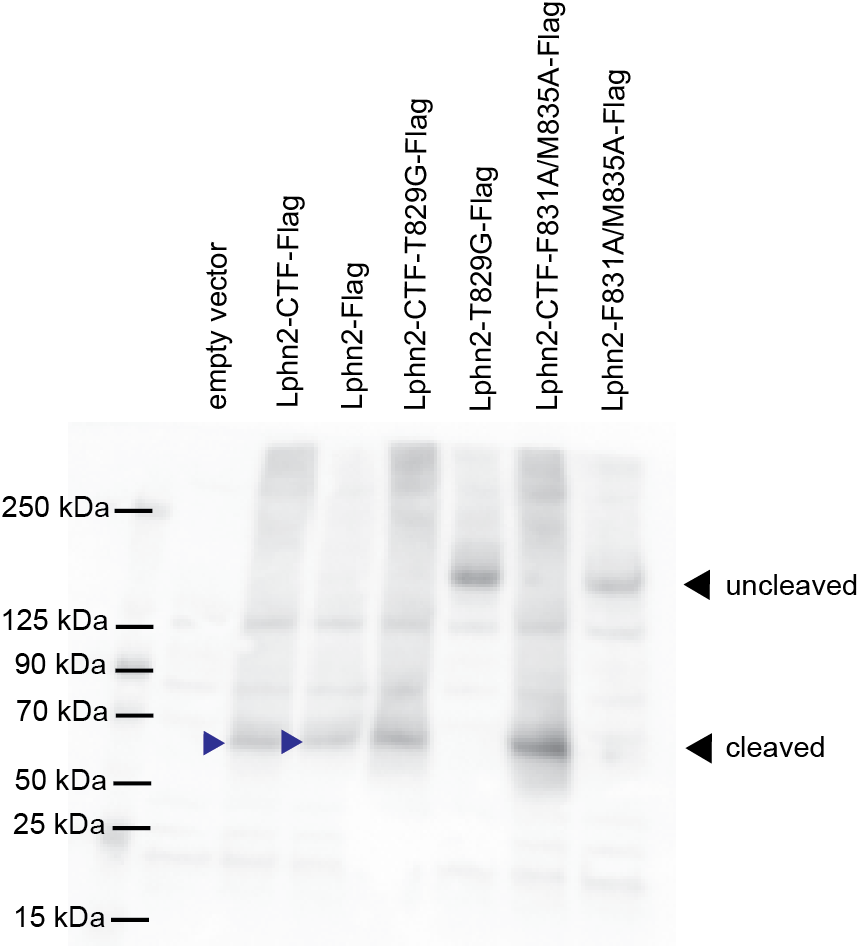

Supplement: Figure 2—source data 2. — Primary antibody against Flag (1:500, ThermoFisher, PA1-984B), secondary antibody anti-rabbit HRP (1:10,000, ThermoFisher, Cat #31458). Blue arrows indicate bands of interest for Lphn2-CTF-Flag and full-length Lphn2-Flag. [file elife-83529-fig2-data2.zip › Figure 2-source data 2/Figure 2 - Source 2.png]

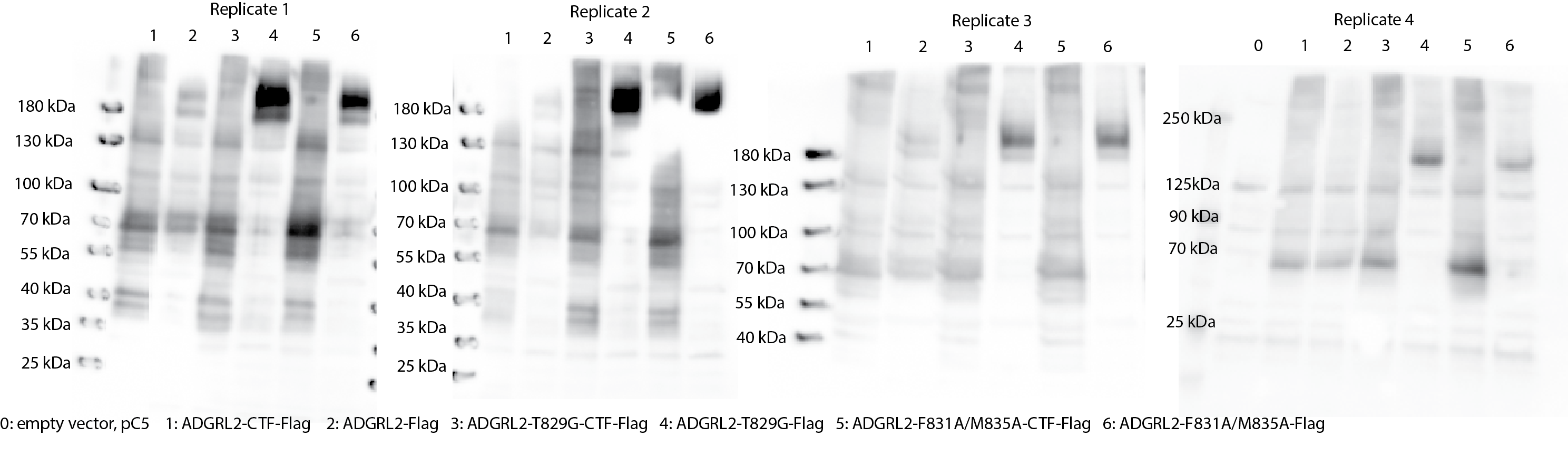

Supplement: Figure 2—source data 3. — Replicate four was used for Figures 2—4. [file elife-83529-fig2-data3.zip › FIgure 2-source data 3/Figure 2 - Source 3.png]

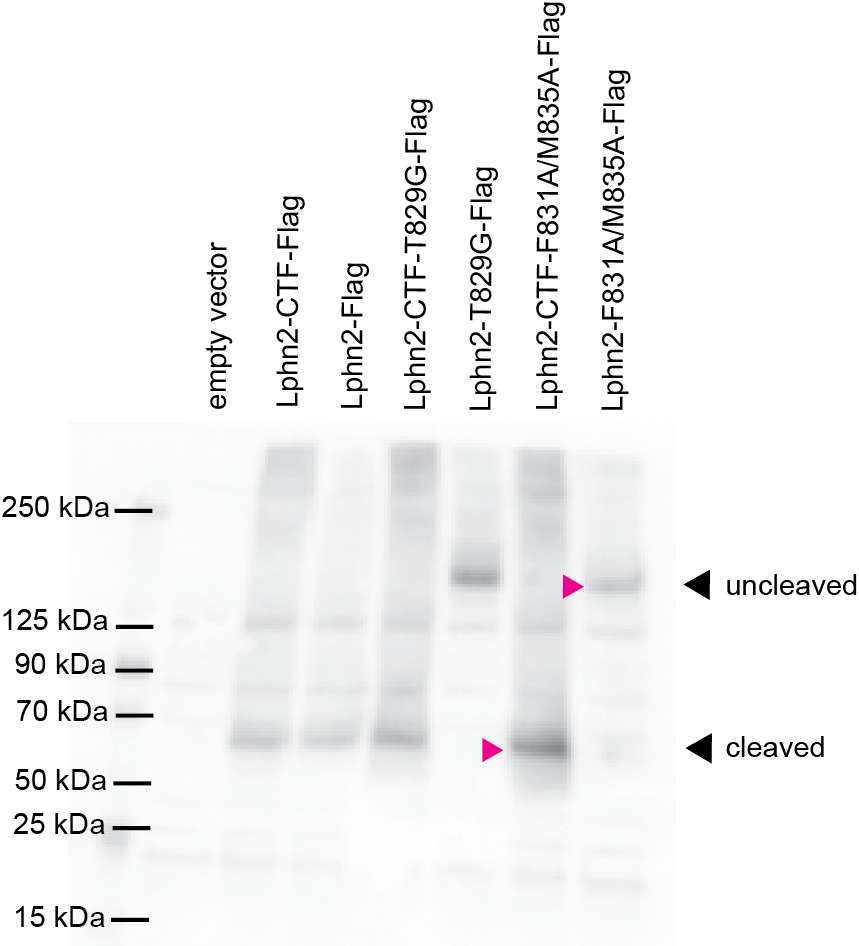

Supplement: Figure 3—source data 1. — Primary antibody against Flag (1:500, ThermoFisher, PA1-984B), secondary antibody anti-rabbit HRP (1:10,000, ThermoFisher, Cat #31458). Magenta arrows indicate bands of interest for Lphn2-F831A/M835A-CTF-Flag and full-length Lphn2-F831A/M835A-Flag. [file elife-83529-fig3-data1.zip › Figure 3-source data 1/Figure 3 - Source 1.png]

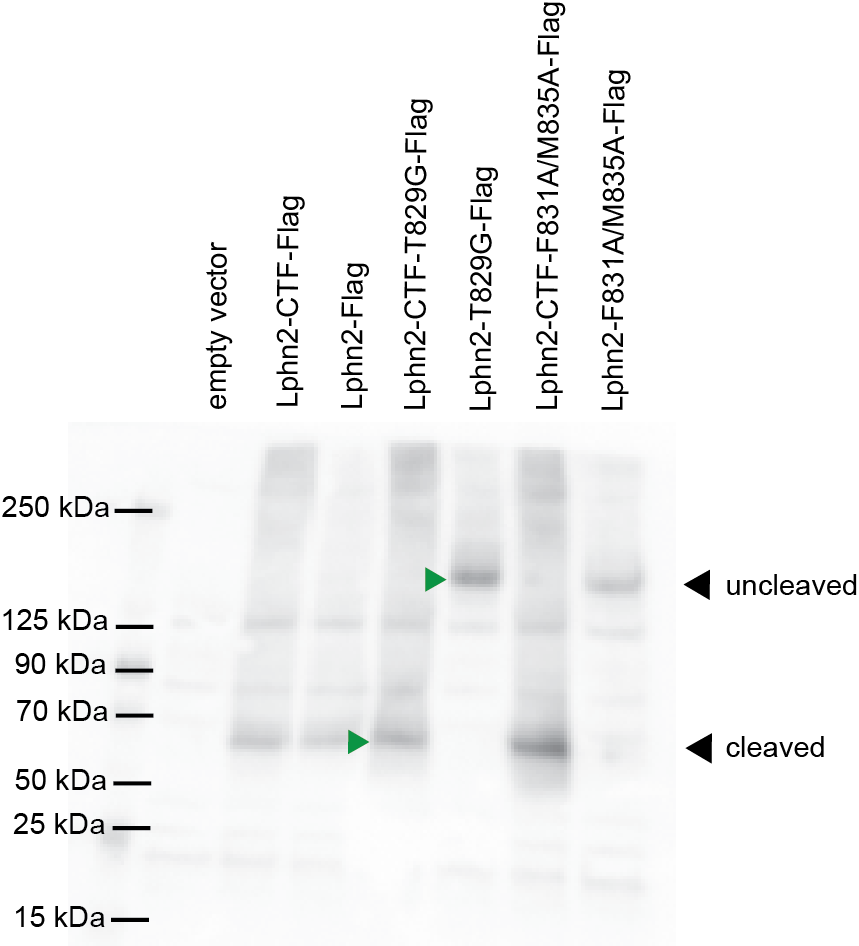

Supplement: Figure 4—source data 1. — Primary antibody against Flag (1:500, ThermoFisher, PA1-984B), secondary antibody anti-rabbit HRP (1:10,000, ThermoFisher, Cat #31458). Green arrows indicate bands of interest for Lphn2-T829G-CTF-Flag and full-length Lphn2-T829G-Flag. [file elife-83529-fig4-data1.zip › Figure 4-source data 1/Figure 4 - Source 1.png]
